# Classifying Low Back Pain Through Pain Mechanisms: A Scoping Review for Physiotherapy Practice

**DOI:** 10.3390/jcm14020412

**Published:** 2025-01-10

**Authors:** Roberto Tedeschi, Federica Giorgi, Daniela Platano, Lisa Berti

**Affiliations:** 1 Department of Biomedical and Neuromotor Sciences, Alma Mater Studiorum, University of Bologna, 40136 Bologna, Italy; daniela.platano@unibo.it (D.P.); lisa.berti@unibo.it (L.B.); 2 Pediatric Physical Medicine and Rehabilitation Unit, IRCCS Institute of Neurological Sciences, 40121 Bologna, Italy; federica.giorgi15@gmail.com; 3 Physical Medicine and Rehabilitation Unit, IRCCS Istituto Ortopedico Rizzoli, 40136 Bologna, Italy

**Keywords:** low back pain, pain mechanisms, nociceptive pain, neuropathic pain, central sensitization

## Abstract

**Background:** Low back pain (LBP) is a leading cause of disability worldwide, often driven by distinct pain mechanisms: nociceptive, neuropathic, and central sensitization. Accurate classification of these mechanisms is critical for guiding effective, targeted treatments. **Methods:** A scoping review was conducted following the Joanna Briggs Institute methodology and reported according to PRISMA-ScR guidelines. A comprehensive literature search was performed in MEDLINE, Cochrane CENTRAL, Scopus, PEDro, and Web of Science. Eligible studies included adults with LBP and focused on clinical criteria for classifying pain mechanisms. Data on study methods, population characteristics, and outcomes were extracted and synthesized. **Results:** Nine studies met the inclusion criteria. Nociceptive pain was characterized by localized symptoms proportional to mechanical triggers, with no neurological signs. Neuropathic pain was associated with burning sensations, dysaesthesia, and a positive neurodynamic straight leg raise (SLR) test. Central sensitization featured widespread pain, hyperalgesia, and disproportionate symptoms. Tools such as painDETECT, DN4, and the Central Sensitisation Inventory (CSI) were validated for neuropathic and central sensitization pain. Central sensitization and neuropathic pain were linked to greater disability and psychological distress compared to nociceptive pain. **Conclusions:** This review aims to provide a historical perspective on pain mechanism classifications and to explore how previous frameworks have influenced current diagnostic concepts in physiotherapy practice. By synthesizing key clinical criteria used to differentiate between nociceptive, neuropathic, and central sensitization pain, this review proposes a practical framework to improve the accuracy of pain classification in clinical settings.

## 1. Introduction

Low back pain (LBP) is one of the most prevalent musculoskeletal conditions worldwide, affecting up to 80% of individuals during their lifetime, with a significant proportion of cases transitioning from acute to chronic pain [1,2]. Chronic LBP, defined as pain persisting for over three months, represents a major clinical and socioeconomic burden, reducing functional independence and quality of life while contributing to work absenteeism and increased healthcare costs [3,4]. Current evidence highlights the importance of recognizing different pain mechanisms—nociceptive, neuropathic, and nociplastic—to guide personalized treatment approaches [2]. Despite its high prevalence, the pathophysiology of LBP is often poorly understood, and clinical management frequently relies on a structural–anatomical paradigm that fails to address the underlying pain mechanisms [5,6,7,8,9].

Pain is now recognized as a complex neurophysiological phenomenon involving peripheral and central nervous system processes. In this context, the mechanism-based classification of pain has emerged as a more accurate framework for understanding and managing LBP, categorizing pain into three primary mechanisms: nociceptive pain, neuropathic pain, and central sensitization [10,11,12]. In addition to these primary pain mechanisms, the drucebo effect, which refers to the negative expectations of patients leading to worsening symptoms or reduced treatment efficacy, has been increasingly recognized as a significant factor in pain management. This effect is particularly relevant in chronic pain conditions, where psychological and emotional components play a substantial role in shaping the patient’s pain experience [12]. Recognizing the drucebo effect as a distinct mechanism allows clinicians to better address the psychosocial factors influencing pain outcomes. Each mechanism is associated with distinct physiological and biochemical processes, which influence both the perception of pain and its clinical presentation.

Nociceptive pain originates from the activation of peripheral nociceptors in response to noxious mechanical, thermal, or chemical stimuli. These stimuli are transduced by specialized ion channels such as TRPV1 (Transient Receptor Potential Vanilloid 1) and propagate as electrical signals through Aδ and C fibers to the dorsal horn of the spinal cord [13,14,15,16]. The release of excitatory neurotransmitters, including glutamate and substance P, activates second-order neurons, which relay nociceptive information to the thalamus and somatosensory cortex for processing [17]. Clinically, nociceptive pain is typically localized, proportional to the stimulus, and associated with tissue injury or inflammation.

In contrast, neuropathic pain arises from injury or dysfunction within the somatosensory nervous system. Mechanisms include abnormal impulse-generating sites (AIGSs), ectopic discharges, and alterations in sodium and potassium ion channel expression, leading to spontaneous neuronal firing and cross-excitation between adjacent nerve fibers [7]. At the central level, neuropathic pain is characterized by structural changes such as the sprouting of Aβ fibers into lamina II of the dorsal horn, where they trigger pain in response to innocuous stimuli (allodynia: refers to a pain response to a stimulus that would not normally provoke pain, typically resulting from altered central pain processing mechanisms) [18,19]. Clinically, patients present with burning, electric-shock-like pain, sensory deficits, and patterns inconsistent with standard dermatomal or myotomal distributions.

The third mechanism, central sensitization, involves an amplification of pain processing within the central nervous system. This is driven by increased excitability of dorsal horn neurons via N-methyl-D-aspartate (NMDA) receptor hyperactivation, neuroinflammation, and the loss of GABAergic inhibition [9,20,21,22]. Dysfunctional descending inhibitory pathways and persistent input from peripheral nociceptors further perpetuate the hyper-responsiveness of central neurons [22,23,24,25]. Clinically, central sensitization manifests as widespread pain, hyperalgesia, allodynia, cognitive dysfunction, fatigue, and symptoms disproportionate to the extent of tissue injury [24,26].

Despite advances in understanding these mechanisms, clinical practice often lacks systematic tools to classify pain in patients with LBP. A failure to identify the dominant pain mechanism can lead to inappropriate or ineffective treatment strategies, particularly in cases of chronic or non-specific LBP [10,11,27,28,29]. Mechanism-based classification offers the potential to guide targeted physiotherapy interventions, including manual therapy, exercise, and neuromodulation, which are tailored to the specific underlying neurophysiological processes [30,31,32].

The aim of this review is to synthesize the current evidence regarding clinical criteria for the mechanism-based classification of LBP. This review will identify key findings from both subjective (patient history) and objective (clinical examination) assessments to propose a clinically applicable framework that assists physiotherapists in distinguishing between nociceptive, neuropathic, and central sensitization pain. By doing so, it seeks to improve clinical decision-making and optimize therapeutic outcomes for individuals suffering from LBP.

## 2. Methods

The present scoping review was carried out following the methodological framework outlined by the Joanna Briggs Institute (JBI) [33] for scoping reviews. To ensure comprehensive and transparent reporting, this study complied with the Preferred Reporting Items for Systematic Reviews and Meta-Analyses Extension for Scoping Reviews (PRISMA-ScR) guidelines [34].

### 2.1. Review Question

We formulated the following research question: “What are the historical and clinical criteria used to classify low back pain according to nociceptive, neuropathic, and central sensitization mechanisms in physiotherapy practice, and how have previous classification frameworks influenced current diagnostic concepts?”.

### 2.2. Eligibility Criteria

Studies were eligible for inclusion if they met the following Population, Concept, and Context (PCC) criteria.

Population (P): Adults (≥18 years) experiencing low back pain (LBP), both acute (≤6 weeks) and chronic (>3 months). Individuals with non-specific LBP, defined as pain in the lumbar region not attributable to a specific pathology (e.g., infection, tumor, fracture, or inflammatory disease). Patients presenting with symptoms suggestive of nociceptive, neuropathic, or central sensitization pain mechanisms.

Concept (C): This review focused on the clinical criteria and assessment methods used to classify LBP based on the underlying pain mechanisms. Included studies explored elements of subjective assessment (e.g., patient history, pain descriptors, and psychosocial factors) and objective assessment (e.g., clinical examination findings, physical tests, and validated tools).

Mechanisms of interest:

- Nociceptive pain: Pain arising from actual or potential tissue damage with identifiable activation of nociceptors.
- Neuropathic pain: Pain caused by a lesion or dysfunction of the somatosensory nervous system.
- Central sensitization: Pain resulting from altered central nervous system processing, including amplified pain perception and reduced inhibition.

Context (C): The context included studies conducted in clinical settings, such as primary care, rehabilitation centers, outpatient physiotherapy clinics, or research laboratories, and studies relevant to the physiotherapy practice or broader rehabilitation contexts, where pain classification informs clinical decision-making. No geographical or cultural restrictions were applied. Only studies published in English were included.

In addition to identifying clinical criteria for classifying pain mechanisms, this review aims to trace the historical evolution of these classifications and examine how previous frameworks have shaped current diagnostic approaches. The methodology was designed to include studies that discussed both clinical criteria and the influence of past classification systems on modern physiotherapy practices. The literature search was broadened to capture historical perspectives, including seminal studies and key developments in pain classification frameworks over time.

### 2.3. Exclusion Criteria

Studies that did not meet the predefined Population, Concept, and Context (PCC) criteria were excluded.

### 2.4. Search Strategy

A preliminary targeted search was conducted in MEDLINE via the PubMed interface to identify studies pertinent to the topic. The indexing terms and keywords retrieved from these initial studies were utilized to develop a comprehensive search strategy for MEDLINE. This strategy was subsequently adapted for other electronic databases, including Cochrane Central Register of Controlled Trials (CENTRAL), Scopus, PEDro, and Web of Science, ensuring coverage of all of the relevant literature. The literature search for this scoping review was conducted between July 2024 and November 2024. The search strategy was finalized on 23 November 2024, ensuring the comprehensive capture of relevant studies published up to that date. The search strings used for each database were as follows:

MEDLINE (PubMed):

(“Low Back Pain”[MeSH Terms] OR “Low Back Pain”[Title/Abstract] OR “LBP”[Title/Abstract]) AND (“Pain Mechanisms”[Title/Abstract] OR “Nociceptive Pain”[MeSH Terms] OR “Neuropathic Pain”[MeSH Terms] OR “Central Sensitisation”[Title/Abstract]) AND (“Clinical Criteria”[Title/Abstract] OR “Physiotherapy Assessment”[Title/Abstract] OR “Pain Classification”[Title/Abstract])

Cochrane Central:

“Low Back Pain” AND (“Pain Mechanisms” OR “Nociceptive Pain” OR “Neuropathic Pain” OR “Central Sensitisation”) AND (“Clinical Criteria” OR “Assessment” OR “Classification”)

Scopus:

TITLE-ABS-KEY (“Low Back Pain” OR “LBP”) AND TITLE-ABS-KEY (“Pain Mechanisms” OR “Nociceptive Pain” OR “Neuropathic Pain” OR “Central Sensitisation”) AND TITLE-ABS-KEY (“Clinical Criteria” OR “Physiotherapy Assessment” OR “Pain Classification”)

PEDro:

Low Back Pain AND Pain Mechanisms OR Nociceptive Pain OR Neuropathic Pain OR Central Sensitisation AND Clinical Criteria OR Physiotherapy Assessment OR Pain Classification

Web of Science:

(“Low Back Pain” OR “LBP”) AND TOPIC: (“Pain Mechanisms” OR “Nociceptive Pain” OR “Neuropathic Pain” OR “Central Sensitisation”) AND TOPIC: (“Clinical Criteria” OR “Physiotherapy Assessment” OR “Pain Classification”)

### 2.5. Study Selection

The study selection process adhered to a structured methodology consistent with scoping review standards. Initially, all search results were compiled and managed using Zotero, where duplicate records were systematically removed. The screening process was conducted in two distinct phases. First, titles and abstracts underwent an initial review, followed by a comprehensive full-text evaluation. Both stages were carried out independently by two reviewers to ensure rigor, with any disagreements resolved through consultation with a third reviewer. The selection process was conducted in alignment with the PRISMA 2020 guidelines, ensuring transparency, reproducibility, and consistency. This systematic approach aimed to identify studies that addressed the research question, thereby supporting a thorough and reliable review process.

### 2.6. Data Extraction and Data Synthesis

The data extraction process was conducted systematically to collect key information from each included study. Extracted details encompassed study design, population characteristics, intervention specifics, outcome measures, and findings relevant to the research question. To ensure consistency, a standardized data extraction form was employed across all studies. For data synthesis, findings were categorized based on specific outcomes, allowing for clear comparisons between studies. A qualitative synthesis was performed to identify recurring patterns, discrepancies, and gaps in the data, providing a comprehensive summary of the evidence. Where applicable, quantitative data were summarized to highlight trends and significant results across the studies. This structured methodology facilitated a clear and methodical synthesis of the available evidence, effectively addressing the research question.

## 3. Results

**Figure 1:**
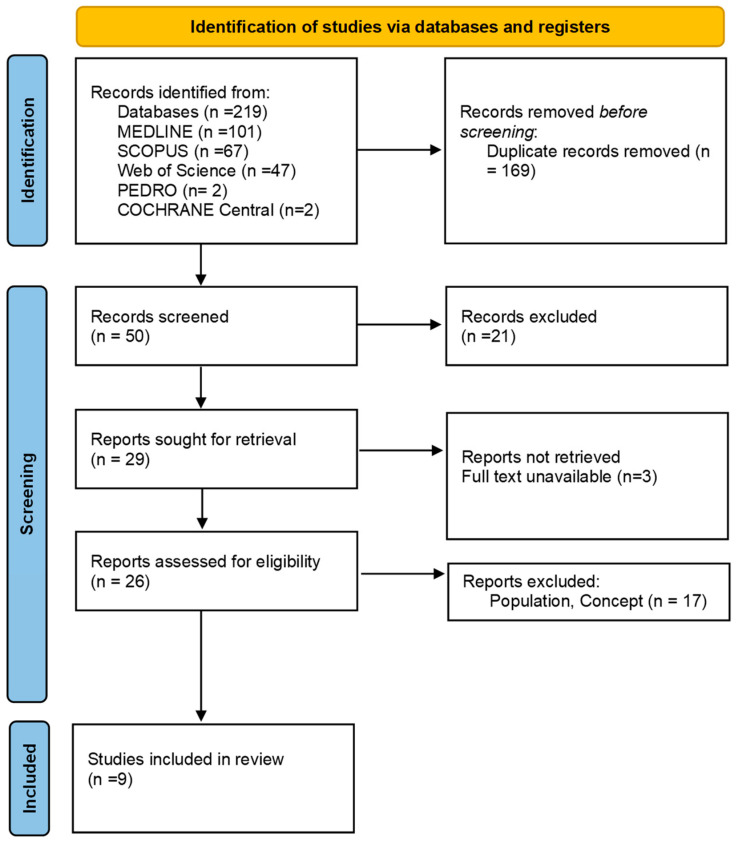
Preferred reporting items for systematic reviews and meta-analyses 2020 (PRISMA) flow chart.

As presented in the PRISMA 2020 flow diagram (Figure 1), from 219 records identified by the initial literature searches, two hundred and ten were excluded and nine articles were included (Table 1). The quality of the studies was assessed in Table 2.

### 3.1. Identification of Nociceptive Pain

Smart et al. (2012c) [38] identified that nociceptive pain is characterized by specific clinical features, including localized pain that is proportional to mechanical triggers, such as movement, posture, or specific activities. These triggers reliably reproduce or alleviate symptoms, making nociceptive pain more predictable compared to other mechanisms. Distinctive features include the absence of neurological signs, such as dysaesthesia, allodynia, or referred pain. Patients in this group often exhibit clear anatomical relationships between pain location and mechanical stressors.

Outcome: The study established clear and reproducible clinical criteria for identifying nociceptive pain, providing physiotherapists with a reliable approach to differentiate this mechanism from neuropathic or central sensitization pain.

### 3.2. Identification of Neuropathic Pain

Smart et al. (2012b) [37] focused on distinguishing neuropathic pain in LBP populations. Key findings included the presence of burning pain, sharp/lancinating sensations, and dysesthesia (abnormal sensations). Positive neurodynamic tests, such as the Straight Leg Raise (SLR), along with symptoms aligning to dermatomal distributions, were identified as critical indicators of neuropathic involvement.

Freynhagen and Baron (2009) [40] further validated the role of diagnostic tools such as the painDETECT questionnaire and DN4 in identifying neuropathic components in LBP patients. Their findings showed a strong correlation between neuropathic symptoms (e.g., radiating leg pain) and positive neurodynamic tests such as SLR.

Beith et al. (2011) [41] added that neuropathic pain is associated with higher pain intensity, greater disability, and psychological distress (including anxiety and depression) compared to nociceptive pain. The study also observed a significant reduction in range of motion (ROM) during passive SLR tests, providing further functional evidence of neuropathic mechanisms. Interestingly, imaging findings such as MRI showed low sensitivity (73%) and specificity (43%) in detecting neuropathic pain, indicating the limitations of relying on structural assessments alone.

Outcome: These studies collectively established robust clinical criteria for neuropathic pain, including specific symptom patterns, neurodynamic test results, and validated diagnostic tools. The correlation between higher disability and psychological distress underscores the importance of early identification and targeted intervention for neuropathic pain mechanisms.

### 3.3. Identification of Central Sensitization Pain

Smart et al. (2012a) [36] reported that central sensitization is characterized by widespread pain, generalized hyperalgesia, and symptoms that are disproportionate to mechanical triggers or identifiable tissue damage. Patients often lack clear anatomical patterns, and their pain presentation includes hypersensitivity and non-mechanical aggravating factors. Using a statistical model based on symptom clustering, the study achieved high sensitivity (91.8%) and specificity (97.7%), validating the key features of central sensitization.

Nijs et al. (2015) [24] proposed a systematic clinical algorithm for identifying central sensitization. This approach integrates subjective findings, such as atypical pain distribution, with objective assessments, including neurodynamic tests and advanced tools such as Quantitative Sensory Testing (QST) and the Central Sensitisation Inventory (CSI).

Sanzarello et al. (2016) [39] further elaborated on the clinical presentation of central sensitization, emphasizing positive neurodynamic tests (e.g., SLR and cross-SLR) and atypical pain patterns as essential indicators. Additional assessments, such as QST and the CSI, were highlighted as valuable tools for confirming the diagnosis of central sensitization in chronic LBP patients.

Smart et al. (2011) [35] added that central sensitization pain lacks mechanical proportionality and is associated with generalized hyperalgesia, widespread pain, and inconsistent responses to physical assessments.

Outcome: These studies demonstrated that central sensitization can be identified through a combination of subjective history, neurodynamic tests, and advanced assessments. Key symptoms include widespread pain, hyperalgesia, and disproportionate responses, providing a clear framework for diagnosis in clinical practice.

### 3.4. Disability and Psychological Impact

Smart et al. (2012) [8] explored the relationship between pain mechanisms and patient-reported outcomes. Patients with central sensitization reported the highest levels of disability, pain intensity, and psychological distress (anxiety and depression) compared to those with neuropathic or nociceptive pain. Measures included the Roland Morris Disability Questionnaire (RMDQ) [42] for disability, the Hospital Anxiety and Depression Scale (HADs) [43,44] for psychological status, and the Short Form-36 (SF-36) [45] for quality of life.

Beith et al. (2011) [41] similarly demonstrated that patients with neuropathic pain experienced higher disability scores, increased psychological distress, and reduced functional capacity compared to patients with nociceptive pain.

Outcome: These findings highlight the significant association between pain mechanisms and functional impairment, as well as the psychological burden of chronic LBP. Patients with central sensitization, in particular, experience more severe disability and mental health challenges, underscoring the importance of a mechanism-based approach to treatment.

### 3.5. Development of Clinical Algorithms and Tools

Nijs et al. (2015) [24] developed an evidence-based clinical algorithm for classifying pain mechanisms in patients with LBP. This algorithm integrates findings from the patient’s history, physical examination, and validated tools such as neurodynamic tests and QST.

Freynhagen and Baron (2009) [40] highlighted the value of diagnostic tools, including painDETECT [46] and DN4, for identifying neuropathic pain. These tools demonstrated strong validity and clinical utility in distinguishing neuropathic components from nociceptive or central sensitization mechanisms.

Outcome: These studies provided practical tools and algorithms to guide clinicians in identifying pain mechanisms, improving diagnostic accuracy, and enabling targeted interventions.

### 3.6. Summary of Results

1. Nociceptive Pain: Localized pain proportional to mechanical triggers with no neurological features.
2. Neuropathic Pain: Burning pain, dysesthesia, positive neurodynamic tests, and higher disability scores.
3. Central Sensitization: Widespread pain, hyperalgesia, disproportionate symptom patterns, and atypical pain distribution.
4. Disability and Psychological Impact: Central sensitization and neuropathic pain are associated with greater functional impairment and higher psychological distress.
5. Clinical Algorithms and Tools: Evidence-based algorithms and tools (e.g., painDETECT, DN4, QST, and the CSI) improve the identification of specific pain mechanisms.

### 3.7. Notes on Tools and Scoring

1. ROBINS-I [47]: Studies with a moderate risk of bias often show issues with confounding variables, missing data, or deviations from the intended interventions.
2. SANRA [48]: Narrative reviews assessed with SANRA may have moderate bias if the literature search is not comprehensive or the justification for conclusions is incomplete.
3. Newcastle–Ottawa Scale (NOS) [49]: For cross-sectional studies, a moderate score is often given when there are minor issues with participant selection or comparability.

## 4. Summary

- The clinical studies show a moderate risk of bias, primarily due to confounding factors, deviations from interventions, or incomplete outcome reporting.
- The narrative reviews generally exhibit a low-to-moderate risk of bias, depending on the thoroughness of the literature search and justification of conclusions.
- The cross-sectional study (Beith et al., 2011 [41]) has a moderate risk of bias due to minor concerns about participant selection and comparability.

## 5. Discussion

The present scoping review aimed to identify and summarize clinical criteria for classifying low back pain (LBP) into three primary pain mechanisms: nociceptive pain, neuropathic pain, and central sensitization. The findings across the included studies offer valuable insights into the distinct features of each pain mechanism, the tools used for their identification, and their relationship to functional and psychological outcomes.

### 5.1. Nociceptive Pain: Clear Patterns and Predictability

The studies by Smart et al. (2012c) [38] and Smart et al. (2011) [35] consistently highlighted that nociceptive pain is characterized by symptoms localized to the anatomical site of tissue injury or mechanical dysfunction. These symptoms are proportional to specific aggravating and alleviating factors, such as movement, posture, or mechanical stressors, and respond predictably during clinical assessments. Importantly, nociceptive pain is associated with the absence of neurological signs, such as dysesthesia, allodynia, or neurodynamic test abnormalities, which are otherwise present in neuropathic pain.

From a clinical perspective, this mechanism can be reliably identified through the patient’s subjective history and objective examination, including postural analysis, palpation, and movement testing. The robustness of these criteria provides clinicians with a straightforward approach for differentiating nociceptive pain from more complex pain presentations. However, it is essential to note that nociceptive pain may coexist with neuropathic or central sensitization mechanisms, particularly in chronic cases, requiring a more nuanced evaluation in such scenarios.

### 5.2. Neuropathic Pain: Unique Sensory Features and Functional Impact

The studies by Smart et al. (2012b) [37], Freynhagen and Baron (2009) [40], and Beith et al. (2011) [41] demonstrated that neuropathic pain has distinct clinical and sensory features that set it apart from nociceptive mechanisms. Key indicators include burning pain, sharp or lancinating sensations, and dysesthesia. Positive findings on neurodynamic tests, such as the Straight Leg Raise (SLR) and its variations, further support the diagnosis of neuropathic pain. These features were consistently aligned with the distribution of dermatomes, indicating an underlying dysfunction or lesion within the somatosensory nervous system.

Additionally, neuropathic pain was associated with greater functional limitations and psychological distress, including higher levels of disability and anxiety/depression (Beith et al., 2011) [41]. The study highlighted reduced range of motion (ROM) during SLR as a functional correlate of neuropathic involvement. However, imaging tools such as MRI were found to have low sensitivity and specificity in detecting neuropathic pain, underscoring the need for a clinical, symptom-based approach.

These findings are clinically significant, as they suggest that validated tools such as the painDETECT and DN4 questionnaires can effectively identify neuropathic components of LBP. This is particularly relevant for clinicians, as patients with neuropathic pain may require interventions beyond conventional mechanical therapies, such as targeted neurodynamic techniques or pharmacological approaches addressing nerve sensitization.

### 5.3. Central Sensitization: A Complex Mechanism with Widespread Implications

Central sensitization emerged as the most challenging mechanism to identify and manage. Studies by Smart et al. (2012a) [36], Nijs et al. (2015) [24], and Sanzarello et al. (2016) [39] emphasized the presence of widespread pain, generalized hyperalgesia, and symptoms that are disproportionate to the extent of tissue injury or mechanical stress. Patients with central sensitization also exhibited hypersensitivity to non-noxious stimuli and inconsistent responses to physical assessments, reflecting the altered pain processing within the central nervous system.

Smart et al. (2012a) [36] validated these findings through a statistical model, achieving high sensitivity (91.8%) and specificity (97.7%) for recognizing central sensitization based on symptom clusters. Similarly, Nijs et al. (2015) [24] proposed a clinical algorithm that integrates subjective and objective assessments, such as pain distribution, neurodynamic testing, and advanced tools such as Quantitative Sensory Testing (QST) and the Central Sensitisation Inventory (CSI). Sanzarello et al. (2016) [39] further underscored the role of neurodynamic tests (e.g., SLR and cross-SLR) and atypical pain patterns in confirming the diagnosis of central sensitization.

From a clinical perspective, recognizing central sensitization is critical because it signifies a maladaptive pain processing state that requires multimodal and patient-centered interventions, such as graded exposure therapy, pain neuroscience education, and cognitive behavioral approaches. Importantly, these patients reported the highest levels of disability and psychological distress (Smart et al., 2012) [8], further underscoring the need for early identification and targeted management strategies.

### 5.4. Clinical and Psychological Outcomes: A Mechanism-Based Perspective

The correlation between pain mechanisms and functional impairment was a key finding across studies. Central sensitization and neuropathic pain were consistently associated with higher levels of disability, as measured by tools such as the Roland Morris Disability Questionnaire (RMDQ), and greater psychological burden, including anxiety and depression (Smart et al., 2012 [8]; Beith et al., 2011 [41]). In contrast, nociceptive pain was linked to lower levels of impairment and more predictable responses to interventions.

Our findings are consistent with more recent studies that highlight the importance of classifying low back pain through a mechanism-based approach to improve patient outcomes [1,2,3]. In particular, the concept of nociplastic pain, recently introduced by the International Association for the Study of Pain (IASP), has refined our understanding of central sensitization and its clinical presentation. Recent studies have proposed new diagnostic frameworks and tools to better identify these pain mechanisms, such as the updated Central Sensitisation Inventory (CSI) and painDETECT, which have shown strong validity in clinical practice [3]. Future research should focus on integrating these tools into personalized pain management strategies to address both the physical and psychological dimensions of chronic low back pain (Fernández-de-Las-Peñas et al., 2023) [4].

These findings highlight the importance of adopting a mechanism-based assessment approach to pain classification. Identifying the dominant pain mechanism enables clinicians to tailor interventions that address the specific neurophysiological processes driving the patient’s symptoms. This approach has the potential to improve clinical outcomes, reduce disability, and alleviate psychological distress.

Beyond the biological and mechanistic understanding of low back pain, it is crucial to consider the social context of individuals experiencing this condition. Social determinants of health, such as socioeconomic status, occupational factors, and support networks, can significantly influence both the experience of pain and the effectiveness of interventions. Future research should focus on integrating social characterization into pain classification frameworks to identify subgroups of patients who may benefit from tailored biopsychosocial approaches. By addressing these social dimensions, healthcare providers can optimize care pathways, improve patient adherence, and enhance long-term outcomes in low back pain management.

## 6. Limitations

This review has several limitations that should be acknowledged. First, the included studies displayed significant heterogeneity in their design, methodologies, and population characteristics, which limits comparability and the strength of evidence synthesis. Second, the absence of a standardized framework for mechanism-based pain classification introduces inconsistencies in identifying and reporting nociceptive, neuropathic, and central sensitization mechanisms. Third, many studies relied heavily on subjective measures, such as patient-reported outcomes and symptom descriptions, which are prone to recall bias and variability in patient perception. Additionally, few studies incorporated objective biomarkers (e.g., neuroimaging or quantitative sensory testing), particularly for central sensitization, limiting the validation of clinical findings.

Furthermore, this review may be influenced by publication bias, as studies with positive results are more likely to be published, potentially skewing the evidence. The predominance of cross-sectional studies restricts causal inferences about the relationship between pain mechanisms and patient outcomes.

Another limitation of this review is the small number of included studies, with only nine meeting the inclusion criteria. This limited pool of evidence may weaken the generalizability of our conclusions. Future systematic reviews should aim to include a broader range of studies to strengthen the evidence base.

## 7. Clinical Practice Implications

The findings of this review provide key guidance for improving the assessment and management of low back pain (LBP) through a mechanism-based approach.

Improved Pain Classification:

Clear clinical criteria for nociceptive, neuropathic, and central sensitization pain, combined with tools such as painDETECT, DN4, and the CSI, enable accurate identification of pain mechanisms.

Targeted Treatment:

o Nociceptive Pain: Responds well to manual therapy, exercise, and posture correction.
o Neuropathic Pain: Requires neurodynamic techniques and adjunct pharmacological therapies.
o Central Sensitization: Demands multimodal interventions such as graded exposure, pain education, and cognitive behavioral approaches.

Addressing Disability and Mental Health:

Recognizing higher disability and psychological burdens in neuropathic and central sensitization pain ensures holistic management that combines physical rehabilitation with mental health support.

Structured Clinical Algorithms:

Implementing algorithms that integrate history, neurodynamic tests, and validated tools enhances diagnostic consistency and guides effective treatment planning.

Optimized Outcomes:

A mechanism-based approach ensures personalized treatments, reducing disability, alleviating pain, and improving overall quality of life for LBP patients.

## 8. Conclusions

This review highlights the importance of a mechanism-based approach for assessing and managing low back pain (LBP). By distinguishing between nociceptive, neuropathic, and central sensitization pain, clinicians can deliver targeted and personalized interventions that address both physical and psychological components.

While the differentiation between nociceptive, neuropathic, and central sensitization pain is already part of clinical practice, this review highlights gaps in the consistent application of these classifications in physiotherapy settings. By implementing validated tools and refining the mechanism-based classification approach, clinicians can achieve more personalized interventions. This refinement is particularly crucial for managing complex chronic pain cases, where traditional approaches may fall short. Addressing these gaps will improve both physical and psychological outcomes for patients.

## Figures and Tables

**Table 1:** Summary of Included Studies on Pain Mechanism Classification in Low Back Pain (LBP).

Author, Year, and Study Type	Methods	Results	Outcomes Achieved
Smart et al., 2011 [35] (Clinical Study)	- Participants: Adults with LBP, with or without referred leg pain.	- Clear discrimination between nociceptive, neuropathic, and central sensitization pain.	Developed clinical indicators to classify pain mechanisms (nociceptive, neuropathic, and central sensitization) in patients with LBP.
	- Exclusion criteria: Diabetes, central nervous system disorders, and pregnancy.	- Nociceptive pain: Localized pain proportional to mechanical stimuli.	
	- Assessment tools: Detailed history and clinical examination, including posture analysis, active/passive movement testing, neurological evaluation, palpation of neural structures, and assessment of symptoms (e.g., allodynia and hyperalgesia).	- Neuropathic pain: Burning pain and positive neurodynamic tests.	
	- Focus: Identification of signs/symptoms distinguishing nociceptive, neuropathic, and central sensitization pain mechanisms.	- Central sensitization: Widespread pain, hyperalgesia, and disproportionate symptomology.	
Smart et al., 2012a [36] (Clinical Study)	- Participants: 452 adults with LBP; 98 met the criteria for central sensitization.	- High sensitivity (91.8%) and specificity (97.7%) for the final model.	Identified key clinical signs and symptoms for central sensitization, with a validated statistical model.
	- Study design: Development of statistical models based on clustering clinical symptoms.	- Key symptoms for central sensitization: Widespread pain, hyperalgesia, absence of clear mechanical triggers, and disproportionate symptom presentation.	
	- Assessment tools: Symptoms categorized into sensitivity clusters, including pain distribution, aggravating/alleviating factors, the presence of hyperalgesia, and functional limitations.		
	- Statistical analysis: Sensitivity and specificity testing of the proposed models.		
Smart et al., 2012b [37] (Clinical Study)	- Participants: Adults with LBP, with or without radiating leg pain.	- Neuropathic pain indicators: Dysaesthesia, sharp/burning pain, positive neurodynamic tests (SLR), and symptoms aligned with dermatomal patterns.	Established clear clinical criteria for identifying neuropathic pain mechanisms in LBP patients.
	- Methods: Development of clinical criteria for neuropathic pain.	- Distinct differentiation from nociceptive pain patterns.	
	- Tools: Patient history (e.g., dysesthesia and burning pain), neurological assessment (SLR test, muscle strength, and reflex testing), and sensory examinations.		
	- Classification: Pain classified as neuropathic or non-neuropathic based on clinical findings.		
Smart et al., 2012c [38] (Clinical Study)	- Participants: Adults presenting with LBP.	- Nociceptive pain characterized by localized symptoms proportional to mechanical triggers.	Developed precise clinical criteria for recognizing nociceptive pain mechanisms in patients with LBP.
	- Methods: Clinical identification of nociceptive pain based on history and a physical examination.	- Absence of neuropathic features (e.g., burning pain, dysesthesia, and hyperalgesia).	
	- Assessment focus: Localized pain, proportional response to aggravating and alleviating factors, and absence of neurological signs (e.g., dysesthesia or allodynia).	- Odds Ratio (OR): 69.79 for identifying nociceptive pain.	
	- Tools: Movement analysis, palpation, and symptom response tracking.		
Smart et al., 2012 [8] (Clinical Study)	- Participants: Same cohort from previous studies.	- Patients with central sensitization showed highest disability, pain, and anxiety/depression scores.	Demonstrated the correlation between pain mechanism classification and disability, quality of life, and mental health outcomes.
	- Methods: Correlation of pain mechanisms with patient-reported outcomes.	- Neuropathic group: Intermediate levels of impairment.	
	- Tools: Questionnaires: RMDQ (Roland Morris Disability Questionnaire), HADs (Hospital Anxiety and Depression Scale), and SF-36 (quality of life).	- Nociceptive group: Least impairment across all measured outcomes.	
	- Clinical classification of nociceptive, neuropathic, and central sensitization pain.		
Nijs et al., 2015 [24] (Narrative Review)	- Objective: Development of guidelines for classifying LBP pain mechanisms.	- Developed a stepwise clinical algorithm for identifying pain mechanisms.	Provided evidence-based guidelines and a clinical algorithm to classify LBP mechanisms for physiotherapy practice.
	- Methods: Reviewed clinical signs, symptoms, and existing tools for distinguishing nociceptive, neuropathic, and central sensitization pain.	(1) Neuropathic: Positive neurodynamic tests, burning pain, and dermatomal distribution.	
	- Focus: Proposing a clinical decision-making algorithm that integrates subjective history (pain distribution and aggravating/alleviating factors) with objective assessments (neurodynamic tests and palpation).	(2) Nociceptive: Localized, mechanical pain.	
		(3) Central sensitization: Diffuse pain, hyperalgesia, and atypical symptom patterns.	
Sanzarello et al., 2016 [39] (Narrative Review)	- Objective: Analysis of central sensitization in chronic LBP.	- Highlighted the importance of recognizing central sensitization through:	Summarized key signs and tools for identifying central sensitization in chronic LBP patients.
	- Methods: A literature review focusing on clinical signs of central sensitization and recommended tools for assessment.	- Atypical pain distribution.	
	- Tools: Patient interview (pain characteristics and atypical distribution), neurodynamic tests (e.g., SLR or cross-SLR), and advanced assessments such as Quantitative Sensory Testing (QST) and the Central Sensitisation Inventory (CSI).	- Neurodynamic testing (SLR or cross-SLR).	
		- Additional tests such as QST and the CSI for confirming the diagnosis.	
Freynhagen and Baron, 2009 [40] (Narrative Review)	- Objective: Identification of neuropathic components in LBP.	- The painDETECT questionnaire effectively identified neuropathic components in LBP patients.	Demonstrated the utility of diagnostic tools (e.g., painDETECT) for recognizing neuropathic pain in LBP.
	- Methods: Review of diagnostic tools (painDETECT, LANSS, and DN4) for detecting neuropathic pain. Clinical signs such as radiating pain, sensory deficits, and positive neurodynamic tests (SLR) were discussed.	- Correlation observed between neuropathic symptoms and radiating pain.	
		- Positive SLR associated with neuropathic involvement.	
Beith et al., 2011 [41] (Cross-Sectional Study)	- Participants: 343 patients with LBP.	- Patients with neuropathic pain reported higher levels of pain, disability, and psychological distress (e.g., anxiety/depression) compared to nociceptive groups.	Correlated neuropathic pain with increased disability and reduced ROM, and highlighted the limitations of MRI in detecting neuropathic involvement.
	- Methods: Patient history, clinical examination (passive SLR and strength tests), and imaging (MRI).	- Reduced ROM in passive SLR observed in neuropathic pain cases.	
	- Tools: Questionnaires: painDETECT (neuropathic screening), NRS (Numerical Rating Scale for pain), and RMDQ (disability).	- MRI showed low sensitivity (73%) and specificity (43%) for neuropathic classification.	
	- Focus: Classification into nociceptive, neuropathic, or mixed pain groups.		

Legend: CSI: Central Sensitization Inventory, DN4: Douleur Neuropathique 4 Questions, HADs: Hospital Anxiety and Depression Scale, LANSS: Leeds Assessment of Neuropathic Symptoms and Signs, LBP: Low Back Pain, MRI: Magnetic Resonance Imaging, NRS: Numerical Rating Scale, OR: Odds Ratio, QST: Quantitative Sensory Testing, RMDQ: Roland Morris Disability Questionnaire, SLR: Straight Leg Raise, SF-36: Short Form-36.

**Table 2:** Risk of Bias Assessment of Included Studies.

Author, Year	Study Type	Tool Used	Key Domains Assessed	Risk of Bias
Smart et al., 2011 [35]	Clinical Study	ROBINS-I	Bias due to confounding	Moderate
			Bias in participant selection	
			Bias in outcome measurement	
Smart et al., 2012a [36]	Clinical Study	ROBINS-I	Bias due to deviations from intended intervention	Moderate
			Bias due to missing data	
			Bias in reporting	
Smart et al., 2012b [37]	Clinical Study	ROBINS-I	Bias in classification of interventions	Moderate
			Bias in measurement of outcomes	
Smart et al., 2012c [38]	Clinical Study	ROBINS-I	Bias due to confounding	Low
			Bias in participant selection	
			Bias in reporting outcomes	
Smart et al., 2012 [8]	Clinical Study	ROBINS-I	Bias in measurement of outcomes	Moderate
			Bias due to missing data	
Nijs et al., 2015 [24]	Narrative Review	SANRA	Justification of the article’s focus	Moderate
			Appropriateness of the literature search	
			Quality of evidence	
Sanzarello et al., 2016 [39]	Narrative Review	SANRA	Relevance of the research focus	Low
			Justification of conclusions	
Freynhagen and Baron, 2009 [40]	Narrative Review	SANRA	Comprehensiveness of literature	Moderate
			Selection bias in included evidence	
Beith et al., 2011 [41]	Cross-sectional Study	Newcastle-Ottawa Scale (NOS)	Selection of participants	Moderate
			Comparability of groups	
			Outcome measurement	
